# The clinically led worforcE and activity redesign (CLEAR) programme: a novel data-driven healthcare improvement methodology

**DOI:** 10.1186/s12913-022-07757-1

**Published:** 2022-03-19

**Authors:** Evelyn J. Corner, Matthew Camilleri, Shruti Dholakia, Charlotte Gredal, Tina Hansen, John Jeans, Marie LeNovere, Jacqueline Mallender, Alex Monkhouse, Claire Purkiss, Tai Ken Ting, Cecilia Vindrola-Padros, Stephen Welfare, Danny Wood, James Wood, on behalf of the CLEAR Faculty

**Affiliations:** 1 33N Ltd, London, UK; 2 Research Dept. of Primary Care and Population Health, University College London, London, UK; 3 Economics By Design, London, UK; 4 Department of Targeted Intervention, University College London, London, UK

**Keywords:** Transformation, Workforce, Innovation, Healthcare, Education, New models of care

## Abstract

**Background:**

The NHS is facing substantial pressures to recover from the COVID-19 pandemic. Optimising workforce modelling is a fundamental component of the recovery plan. The Clinically Lead workforcE and Activity Redesign (CLEAR) programme is a unique methodology that trains clinicians to redesign services, building intrinsic capacity and capability, optimising patient care and minimising the need for costly external consultancy. This paper describes the CLEAR methodology and the evaluation of previous CLEAR projects, including the return on investment.

**Methods:**

CLEAR is a work-based learning programme that combines qualitative techniques with data analytics to build innovations and new models of care. It has four unique stages: (1) Clinical engagement- used to gather rich insights from stakeholders and clinicians. (2) Data interrogation- utilising clinical and workforce data for cohort analysis. (3) Innovation- using structured innovation methods to develop new models of care. (4) Recommendations- report writing, impact assessment and presentation of key findings to executive boards. A mixed-methods formative evaluation was carried out on completed projects, which included semi-structured interviews and surveys with CLEAR associates and stakeholders, and a health economic logic model that was developed to link the inputs, processes, outputs and the outcome of CLEAR as well as the potential impacts of the changes identified from the projects.

**Results:**

CLEAR provides a more cost-effective delivery of complex change programmes than the alternatives – resulting in a cost saving of £1.90 for every £1 spent independent of implementation success. Results suggest that CLEAR recommendations are more likely to be implemented compared to other complex healthcare interventions because of the levels of clinical engagement and have a potential return on investment of up to £14 over 5 years for every £1 invested. CLEAR appears to have a positive impact on staff retention and wellbeing, the cost of a CLEAR project is covered if one medical consultant remains in post for a year.

**Conclusions:**

The unique CLEAR methodology is a clinically effective and cost-effective complex healthcare innovation that optimises workforce and activity design, as well as improving staff retention. Embedding CLEAR methodology in the NHS could have substantial impact on patient care, staff well-being and service provision.

**Supplementary Information:**

The online version contains supplementary material available at 10.1186/s12913-022-07757-1.

## Contribution to the literature

• Optimisation of healthcare workforce and systems improves patient safety, patient outcomes, staff retention and cost effectiveness and is a core part of the United Kingdom’s National Health Service recovery plan.

• The CLEAR methodology is more than just process redesign- it teaches clinicians how to use clinical insights and locally collected data to deliver bespoke efficient and effective workforce and system redesign. This builds intrinsic capacity to iteratively improve systems led by the existing workforce

• CLEAR is sponsored by Health Education England (HEE) and has been commissioned by both HEE and NHSEI. The programme has been rolled out across five national themes, including critical care, mental health, anticipatory care, urgent and emergency care and ophthalmology. This is the first paper describing the CLEAR methodology in detail, which will support stakeholder understanding of the programme.

• Embedding the methodology through a national faculty will provide an economically sustainable approach to achieve workforce redesign. This improves patient care and outcomes empowering staff to transform care in a tangible and inclusive manner, while building a range of leadership and research skills.

• The CLEAR methodology has been shown to deliver a cost efficiency of at least £1.90 for every £1 compared with outsourcing. Results suggest CLEAR recommendations are more likely to be implemented compared to other complex healthcare interventions because of the levels of clinical engagement and have a potential return on investment of up to £14 over 5 years for every £1 invested. This paper will increase understanding of the CLEAR approach and facilitate the development of regional CLEAR hubs and faculty, with the potential to change the NHS approach to workforce and activity redesign substantially.

## Background

### Context

In 2017/18 the NHS spent £26.6 million on private healthcare consultancy [1], however many consultancy firms have been criticised for lacking the technical expertise in clinical care which is necessary to develop appropriate innovations and effect change in healthcare workforce and systems redesign [2, 3]. CLEAR, which stands for Clinically Led workforcE and Activity Redesign [4], is a unique programme which evolved from health and care transformation work in northwest England and from the personal experiences of the clinical staff who devised and led it.

These clinicians were all relatives of patients who experienced the impact of suboptimal care due to system (not clinician) failure and all made the decision to temporarily leave specialist training because of this. Their subsequent experiences were varied, including working with a private workforce redesign initiative, a paediatric intensive care unit and performing an activity-driven, workforce redesign in a hospital in Yangon, Myanmar. Coming together, they reflected on the importance of clinical and workforce data to support any redesign initiative, regardless of where in the world this initiative was undertaken. Extracting these data is challenging, but with the appropriate Information Governance (IG) and technical support, the conversion of clinical databases into usable dashboards would ensure that services could be designed around the local needs of the patients. In addition, training clinicians on big data analytics, who also have the clinical knowledge to meaningfully interpret it, could transform the way that service redesign is approached within the NHS. This, with input from East Lancashire NHS Trust, led to the idea for CLEAR, which was then piloted in seven sites for urgent and emergency care across England in 2019.

The essence of the CLEAR collaborative approach is bringing together real experiences and knowledge of frontline clinical staff, with innovative analytical, visualisation, and data modelling, to create evidence-based transformational change led by clinicians that understand the health needs of the population. This could radically transform health service reform empowering staff to redesign services more efficiently in a way that aligns to local needs, whilst significantly reducing consultancy costs to the NHS and improving patient outcomes.

### Purpose

This article presents the CLEAR programme and unique methodology which is commissioned by Health Education England (HEE), and NHS England and NHS Improvement and delivered by East Lancashire Hospitals Trust and 33n Ltd., a private, clinically led healthcare, education and analytics firm of clinicians, data engineers and scientists.

The purpose of CLEAR is to empower clinicians to improve patient outcomes and staff wellbeing through clinical engagement and data-led innovation. CLEAR comprises four unique steps, that: (1) collate qualitative data from clinical engagement, and locally collected clinical and workforce quantitative data, (2) provide dashboards that visualise these locally available clinical data sets, (3) allow participating staff to understand the root cause of health service and workforce issues, (4) triangulate data to drive innovation and workforce redesign and (5) bring together service leaders and clinical sponsors to ensure that the solutions are pragmatic, implementable and grounded in the data.

This paper will describe the CLEAR methodology in detail as well as present the findings from a mixed method and health economic evaluation of the CLEAR programme.

## Methods

### The CLEAR approach

CLEAR is a 22–26-week (theme-dependent) apprenticeship style methodology currently with five national themes: urgent and emergency care, mental health, anticipatory care, critical care and ophthalmology. The methodology respects the nuances of each theme through its design, ensuring that the approach is transferable to achieve successful outcomes in that area. There are 4–8 participating organisations per theme at any one time, and 2–4 clinicians per organisation, who are seconded to the CLEAR Faculty as associates. The inclusion of multiple organisations and associates serves to create cross-system learning and broadens the range of insights developed.

CLEAR themes are typically sponsored by national bodies, such as Health Education England (HEE) and NHS England and NHS Improvement (NHSE&I), to address strategic priorities for the NHS as outlined within the NHS Long Term Plan [5]. For nationally sponsored themes, a competitive expression of interest (EOI) process gives organisations equal opportunity to apply for participation. CLEAR projects may also be sponsored at a regional or system level to address local priorities.

For national themes interested sites firstly complete an EOI form online. In order to be selected they have to show that they are able to meet site initiation timelines, which includes a memorandum of understanding, information governance sign off, data preparation and identification of a ‘clinical’ and ‘executive sponsor’.

The clinical and executive sponsors work with the CLEAR faculty to refine the scope of the work, ensuring that it addresses locally relevant issues and is aligned to their strategic objectives. Once the scope has been refined they help to champion the project within the trust, supporting identification of the correct individuals to engage with including leads for clinical areas, information governance and business intelligence. In addition, executive sponsors communicate the work at executive level to ensure implementation and approve appropriate release of clinicians to perform the education and delivery. Clinical sponsors are more involved in the project delivery, supporting the associates on the ground and promoting the project within the clinical team.

The inclusion of executive and clinical sponsors helps to ensure the process of codesign, and empowerment of frontline clinicians- ensuring that solutions are more likely to be implemented.

The CLEAR apprenticeship model includes an in-depth, blended learning element which runs in parallel to the live project. The education is delivered using a combination of online learning platforms: Blackboard (https://www.blackboard.com/en-uk), Panopto (https://www.panopto.com/) and miro (https://miro.com/online-whiteboard/). These were used to create online workbooks and pre-recorded lectures as well as remotely delivered live workshops and tutorials, and where possible face-to-face sessions. Associates are provided with learning outcomes and are guided through the education and live delivery by team supervisors and CLEAR Faculty. The educational package is designed to equip associates with the knowledge, skills and experience to deliver a successful CLEAR project - this also builds into a career pathway for CLEAR associates, taking them through from the foundations of CLEAR to associate, fellow, and finally practitioner level. An example of the learning outcomes can be found in the supplementary material (Appendix 1).

**Fig. 1:**
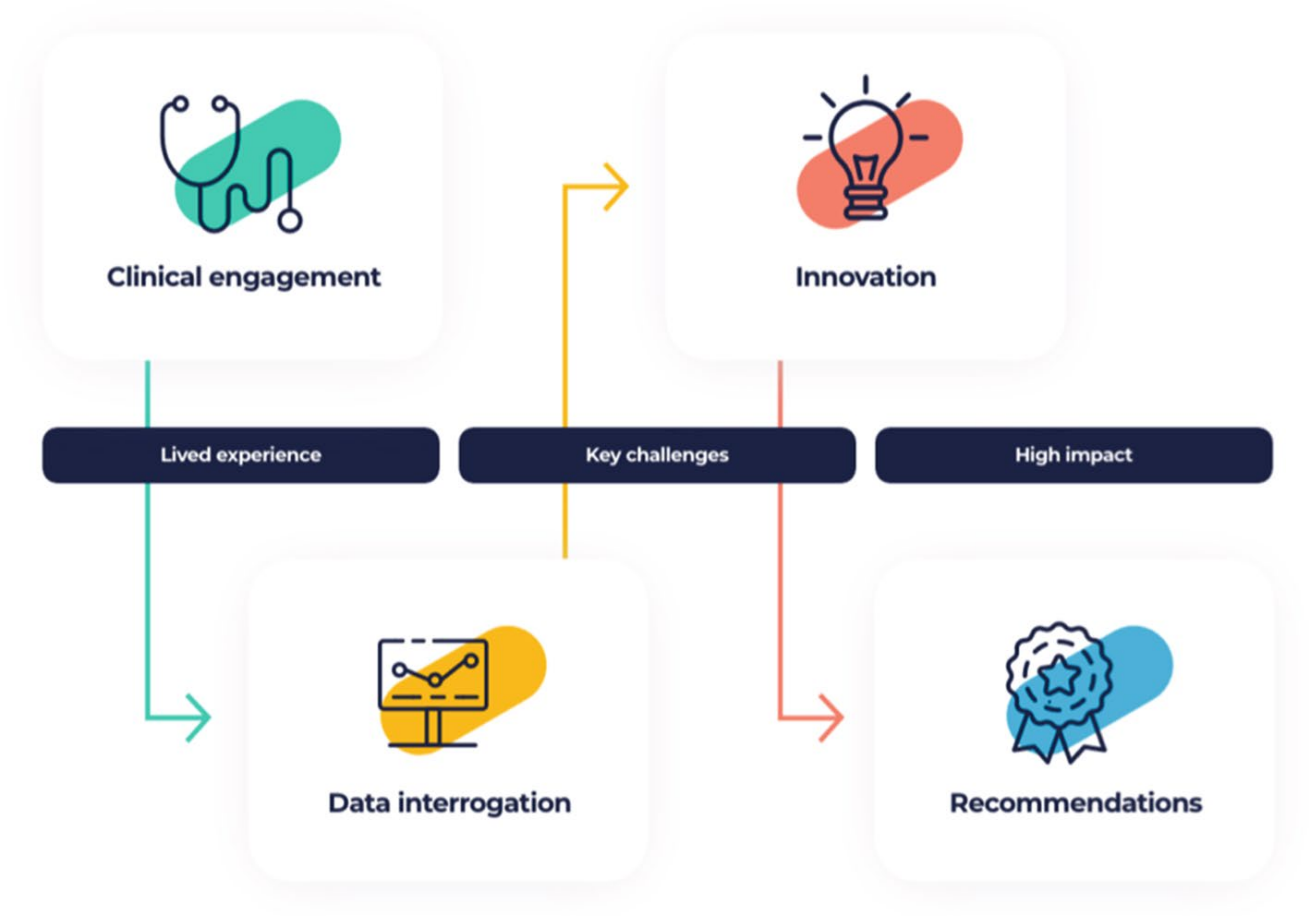
The four stages of CLEAR

High levels of engagement with clinical and executive sponsors in addition to the legacy of the education ensures that this programme is more than pure process redesign. The four stages of CLEAR are described and set out in the diagram below (Fig. 1).

#### Stage 1: Clinical engagement (weeks 0–8)

During the clinical engagement stage, associates identify key stakeholders involved in leading, delivering and interacting with the service. This is supported by the clinical sponsor. A stakeholder register, comprising a purposive sample of staff working in the area/service, is identified for the CLEAR associates to engage with- this includes, but is not limited to, service leads, clinical staff e.g., nurse, doctors and allied health professionals, and non-clinical staff, e.g., porters and administrators.

Engagement involves using a series of qualitative techniques, such as interviews, focus groups, field observations and informal discussions. The goal of these engagements is to collect rich data about the service, such as patient pathways, workforce issues, staffing, problematic cohorts and staff well-being. This gives associates a rich description of how the system functions, and the staff perceptions of what works well and why, and what needs improving. The purposive sampling approaches allows CLEAR associates to collect dissonant views which highlights contrasting opinions and provides a holistic picture. Ideally, associates aim for data saturation, which means that no new themes are emerging from the data. Typically, a CLEAR team conduct around 30–40 engagements.

Engagements are recorded with an automatic transcription function on Panopto. These qualitative data are then recorded in a paraphrased fashion onto a clinical engagement tool, which is an Excel spreadsheet that allows central recording of data collected by all associates involved in the project. A rapid thematic analysis approach is used to analyse the data. The steps to the thematic analysis are outlined below:

- **Step 1**: Each associate completes 2–3 engagements each. They record them on the clinical engagement tool and then apply short descriptive codes. This is done independently.
- **Step 2:** Associates meet, merge the data into one central tab and agree the master codes for ongoing analysis by condensing individual codes that emerge.
- **Step 3:** Associates continue to collect data and code it by applying the master code framework, whilst also developing and agreeing additional codes as required.
- **Step 4**: Once the open coding is complete i.e., all data is coded, family codes are formed, which are a collection of interconnected ideas.
- **Step 5:** Family codes are developed further into categories and finally key themes that are presented back to the *clinical sponsors*.

The themes are prioritised by the team and the clinical sponsor, and the most pressing undergo root causes analysis using fishbone diagrams [6]. This process allows associates to identify the perceived issues that contribute to the key themes and present them in a visual manner to be easily interpreted by the team. This is followed by the development of c*ausative statements*, which are statements that link the key issues, with the cause of the issue and the effect that issues have on the service. These statements create surrogate hypotheses which can be taken forward to the next stage of the CLEAR methodology, data interrogation (see Appendix 2 in the supplementary material for a worked example).

#### Stage 2: Data interrogation (8–14)

Data interrogation involves in-depth analysis of clinical and workforce data of organisations. The data used, and the way it is visualised for the purpose of interrogation are unique stages in the CLEAR methodology, and therefore the process of accessing data and visualisation of that data warrants further discussion prior to consideration of the interrogation approach.

### Scoping

The scope of the work is first determined with the site with the clinical and data teams working together to create a specification that will capture the required available data for successful completion of the project. The information governance documentation, which is completed as part of the contracting stage (refer below) reflects this data specification. Where possible, the data specification is aligned with nationally submitted datasets such as the Emergency Care Data Set (ECDS) and the Mental Health Services Data Set (MHSDS), which allows standardisation of the data requested between different sites. The objectives of an exploratory interrogation of the data sets means that the process of determining data specification uses hypothesis driven modelling of what data fields might be relevant and useful to answer key clinical questions across a broad range of data sets and clinical domains. The process of local clinical validation of data that is extracted also supports the organisation with improving the quality of this data submission with NHS Digital.

### Information governance

Prior to any data processing, appropriate and compliant data sharing documentation – including a data processing impact assessment and a sharing agreement – are collaboratively drafted by 33n and the participating organisation. Draft documents are reviewed for approval by senior stakeholders, from both organisations, with responsibility for information governance and data protection.

33n completes the NHS Digital Data Security and Protection Toolkit [7] assessment every year and is Cyber Essential Plus certified. Data shared from a trust’s systems is stored on private, encrypted, access restricted servers in the London region. All data are deidentified i.e., a data subject cannot be directly identified by any member of the 33n team. This means that no NHS numbers, names, residential address, etc., are included.

### Data collection

The data required for the projects supports the understanding of the patient activity and the workforce availability at the organisation. Patient activity data is gathered from the patient administration system (PAS) and supplementary sources, such as national audit data. This data describes details of patient referrals, contacts and attendances, including their demographics, reasons for attendance, movements through the organisation, diagnoses, procedures and outcomes. Brought together, this builds a detailed, granular picture of the requirement for care within the organisation and how that care is being delivered in a way that has not been done before. The workforce data is brought together from a combination of electronic staff record (ESR) and finance data. This describes the workforce that delivers care by whole time equivalent (WTE) per role and outlines the monthly spend for substantive, bank and agency requirement. The workforce data provides an important baseline understanding of the workforce available within the organisation to deliver care for patients.

The types of data that feed into the dashboards will vary dependent on the scope of the project. This broadly falls into the following categories, shown in Table 1.

**Table 1:** Types of clinical and workforce data

Data type	Data category	Data field examples
Patient activity data	Demographic data	Gender, racial/ethnic origin, age, Lower Layer Super Output Codes (LSOA), GP practice code, etc.
	Clinical data	Patient investigations, diagnosis, and treatment data: vital signs, patient observations, investigation types and results, medical specialty, etc.
	Patient flow data	Patient attendance, referrals, admissions, outcomes, time stamps, locations, type of contact etc., including patient and episode numbers (or equivalent)
	Clinical coding data	Relevant clinical classifications and coding (diagnosis, procedure, consultant codes, frailty, clustering codes, etc).
Workforce data	Workforce composition data	Volume and types of staff position and roles, qualifications, rota details, contracted hours, shift time, etc.
	Finance data	Staff. bank and agency spend, locum spend, administrative costs, etc.

The data highlighted in Table 1 are accessed via established databases, such as: electronic staff records, electronic patient activity records/systems, ward movement databases, test request systems, national audit data, roster data, financial systems, and theme specific data sets, such as The Mental Health Services Data Set (MHSDS), and The Emergency Care Data Set (ECDS).

In most instances, the data required is extracted from the organisation by local Business Intelligence (BI) teams who have an in-depth knowledge of the organisation’s systems and software. Three consecutive years of data are requested to obtain a historical view of the activity and how this has changed over time. Once the data has been extracted by the organisation’s BI team, it is then transferred via a secure upload to 33n’s London-based servers for processing.

### Data processing and visualisation process

**Fig. 2:**
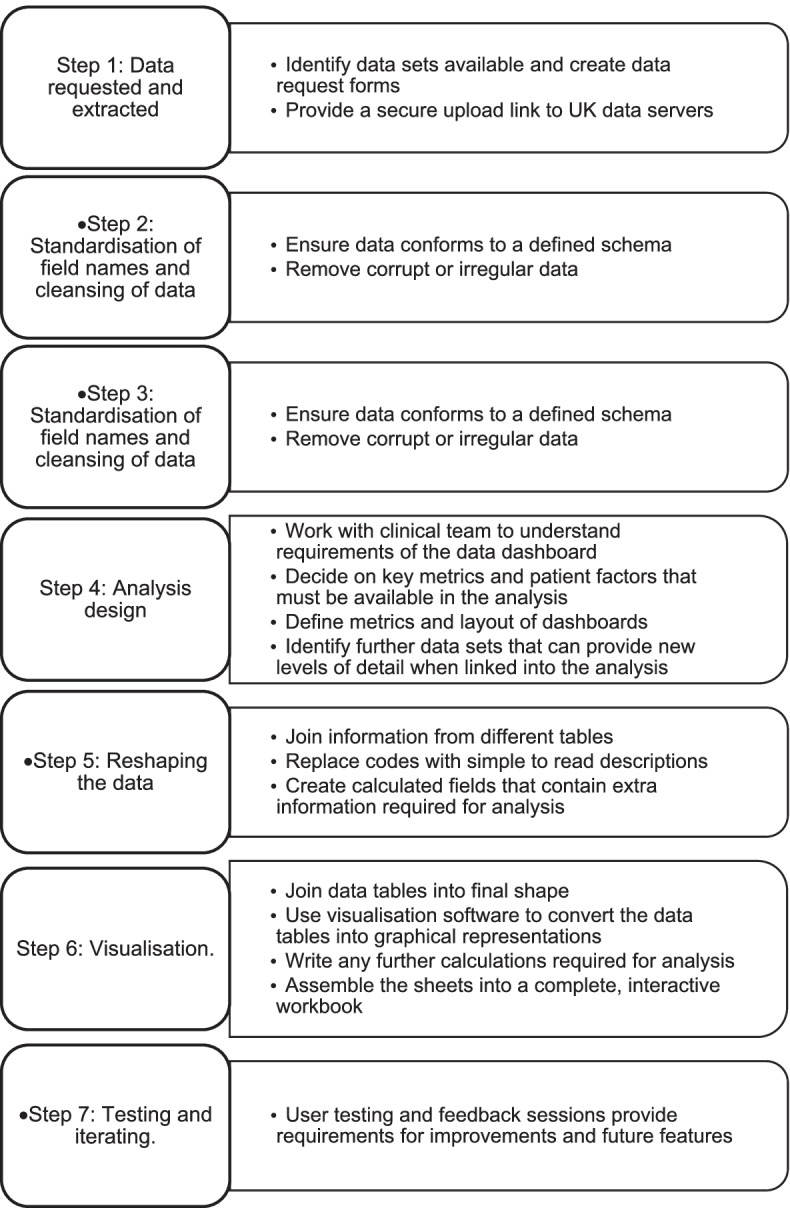
Data processing

Data processing is required to transform the data from its raw form into tables from which usable dashboards may be built. This includes a series of validation and transformation steps outlined in Fig. 2. A worked example of data processing for a UEC project is outlined in Appendix 3.

### Data interrogation process

Quantitative data interrogation is completed using Tableau™ Software (version 2021.3) (LLC). Tableau™ allows data to be visualised in easy-to-read dashboards and visualisations that allow quantitative clinical data to be combined in multiple layers. The ability to apply multiple filters to the data provides insight into healthcare metrics of performance and the clinical pathways and processes that impact clinical departments or systems. Quantitative data visualised in Tableau™ is site specific: no other healthcare provider’s data is included in Tableau™. A UEC (Urgent and Emergency Care) Tableau™ would not, for example, include quantitative data shared by a primary care healthcare provider.

The associates are given access to Tableau™ and advised to complete an exploratory interrogation of the data as whole. This gives insight into the CLEAR site’s metrics of performance; for example, in the case of urgent and emergency care, the number of referrals or attendances, patient flow, large cohort groups that use the service, and discharge destination or attendance conclusion. These data can be viewed by diagnostic groups, investigations, interventions, and locations. Patterns of activity and demand can be visualised and data can be compared against national Key Performance Indicators (KPI’s) or quality standards to give a benchmark of how the site compares against expected measures of performance.

The associates then apply agreed filters to the data to interrogate cohorts of interest or processes that have been identified via one of three methods (1) cohorts of interest identified by the CLEAR site in the initial scope document, (2) cohorts of interest identified during the clinical engagement phase (via coding of data and fishbone diagrams), and finally (3) cohorts that have arisen during the initial quantitative interrogation of the data described above. The filters used to create cohorts for interrogation are agreed as a CLEAR site team, and recorded on the data interrogation tool, to ensure that all associates belonging to the team are using identical filters ensuring data hygiene.

Cohorts can then be interrogated in further detail to review how they affect the performance of the CLEAR site e.g., size of the cohort, does the cohort have multiple pathways, does the KPI data vary for specific cohorts and what is the impact on the department.

Associates record their data findings on a data interrogation tool, which captures descriptive statistics, screenshots of data visualisations and short statements describing what the data shows and whether this links to the qualitative data.

At the end of the quantitative data phase, the associates triangulate (8) the qualitative and quantitative data. Associates create theoretical frameworks or hypotheses to explain the potential causes of key challenges that arose during the clinical engagement phase and then use the qualitative and quantitative data to support or refute these hypotheses.

The triangulated data should provide detailed information of the root cause of site challenges providing a clear starting point for the innovation phase that comes next.

Appendix 2 in the supplementary material includes examples of the Tableau™ data dashboard and a worked example of the triangulated data from a UEC project.

#### Step 3: Innovation (weeks 15–20)

During the innovation phase, associates are encouraged to use divergent thought processes to look for new and innovative solutions. The aim is to enable second order change through the design of a new model of caring for patients, and new ways of staffing through workforce redesign. The associates are provided with several tools for the creation, refinement and impact assessment of their ideas and solutions to the challenges highlighted through the triangulation of the qualitative and quantitative data [8].

Tools for generating multiple and varied ideas are demonstrated and given to the teams to use, such as ‘fresh eyes’ and ‘steppingstones’ [9]. After the use of these tools, there will be many options of varying plausibility and viability for conceptual solutions to the challenges.

The ideas generated then need to be looked at with a more convergent thought process to develop the concepts into workable solutions. The teams are provided with further tools on idea refinement including ‘dot voting’ [9] and linking or grouping solutions. This allows teams to select the concepts most likely to be successfully implemented but also to build on concepts by linking ideas into larger more coherent plans for change. At the end of this process, teams should have selected one or two solutions for each challenge and developed the concepts into more workable solutions.

The teams then perform an impact assessment of their solutions. This has a two-fold intention helping to further refine the ideas and solutions, but also select the most favourable options to put forward as recommendations. Tools provided for this include Levitt’s Diamond [9], Yesterday Tomorrow [9] and use of an ease of implementation versus desirability matrix.

By the end of these exercises, the teams should have new models of care developed as solutions to the challenges with a stratification of their ease of implementation against them.

A key part of ensuring success of an innovation is securing agreement of stakeholders to the issues and findings and socialising the innovations early so that they can be made as robust as possible for the recommendations stage. This is carried out through stakeholder meetings during the programme.

The next stage is the designing a workforce to the new processes.

### Workforce

Workforce redesign is an integral part of the innovation phase as it helps to consolidate the understanding of the challenge with the innovative new solutions. By working collaboratively with the local stakeholders, new models of care and workforce are designed to improve patient care and empower staff whilst being pragmatic and sustainable.

To achieve this outcome, a workforce methodology based on well-established concepts of healthcare demand and capacity modelling [10, 11] is utilised with the following considerations:

- Describe the target cohort of patients as reviewed through the qualitative and quantitative analysis.
- Understand in detail the target cohort characteristics from demographic factors, attendance behaviour, patient journey, activity and outcome generated during their interaction with the service.
- Describe the new model of care for these patients generated from the innovation phase. This requires a thorough understanding of the new patient pathway and the intended aims of the new service.
- Describe the patient demand, as characterised by attendance and activity demand. This is described as the care required per patient by each type of workforce, per location for every hour of every day. This allows for a flexible workforce model that expands and constricts to meet the varying demand of peak and off-peak hours in an operating service, whilst considering official guidelines of 80th centile of attendances [12]. By reviewing patient care requirements through activities generated, a skills-based approach can be engaged to address these needs. This promotes innovative new roles to meet demand through upskilling or cross-skilling across professions.
- Describe the workforce capacity in this new model. Various considerations can be employed here including estates capacity, minimum staffing requirements, safer staffing targets, workforce efficiency and local variations in roster patterns.
- Match patient activity and attendance demand to workforce capacity to identify the appropriate, sustainable and safe effective workforce in the new model of care.

The teams will build a series of potential workforce models to deliver the new model of care that has been designed. These form different options that may be presented for consideration. For example, one option may reflect the utilisation of a new role which can then be compared to more traditional models.

The workforce data extracted from the site is used as a baseline of current staffing and spend. The new models of workforce are compared against the baseline to understand the change in workforce profile that would be required to staff the new model of care. The financial implications of these models are calculated using the NHS contract payscales (AfC, DDRB and GMS) estimated on-cost of 20% [13, 14].

The approach, as described above, emphasises a ground-up, patient-demand based workforce model which takes into consideration multiple demand and capacity variables along the way. The final workforce model is bespoke to the local team creating it, emphasising that solutions need to be clinically-led, supported by data and tailored to the local context. These workforce models are an important part of the recommendations for the project.

Appendix 2 in the supplementary material contains examples of the workforce modelling tool output and the implementation versus desirability matrix.

#### Step 4: Recommendations (weeks 20–24)

The projects close with the generation of recommendations for change, in which the team brings together the work that has been completed in the previous stages and synthesise a clearly articulated case for change. The recommendations are written as a series of options which vary in their ease of implementation and investment. The impact of each option is described in terms of the anticipated change in workforce, process improvement, financial cost, KPIs, staff and patient experience, along with a suggested implementation roadmap that includes the relevant metrics to evaluate the impact of the solutions. A worked example of a project implementation roadmap and recommended metrics is shown in Appendix 2 of the supplementary material.

The project team present the recommendations to the executive board for consideration and produce a written report that may be circulated to stakeholders. If the recommendations are accepted by the executive board, the written report may be used by the site to develop a business case to support implementation. In the post project phase contact is kept with the site to track and support the implementation of the recommendations and at mutually agreed points further evaluation of the impact of any implemented solutions are undertaken with a refresh of the data to help facilitate this.

The implementation of recommendations is at the discretion of the participating organisation and is locally owned. However, as part of the final report, project associates are required to set out a site-specific high -level implementation roadmap including key time-based sequence of activities, key stakeholders and any process or estate considerations. A suggested implementation strategy is included within the written report, including recommended outcome metrics and re-evaluation time scales.

### Evaluation of CLEAR

CLEAR programmes have undergone external evaluation to determine a potential return on investment.

The purpose of the evaluation was to:

1. Assess the extent to which CLEAR projects deliver on the value promise and achieves the core aims of the programme
2. Assess the return on investment (RoI) a CLEAR project may bring to a participating NHS organisation and sponsors
3. Inform the future direction and development of CLEAR (not presented here).

A formative evaluation methodology was used, which included a qualitative study followed by an economic evaluation that the qualitative data helped to inform. A health economic logic model was developed to link the inputs, processes, outputs and the outcome of CLEAR as well as the potential impacts of the changes identified from the projects.

The data on which the analysis was performed included:

- Interviews with people involved in the design of CLEAR (*n* = 4) and previous CLEAR programme associates (*n* = 6)
- Interviews with people who have been or are currently CLEAR delivery or education leads (*n* = 5)
- Survey with previous CLEAR programme associates (*n* = 14) Reports and recommendations from previous CLEAR projects 7
- The health economic logic model
- Reports from previous CLEAR projects

#### Health economic logic model

The cost of each CLEAR project was calculated based on information provided by 33n Ltd. about each of the components of a CLEAR project. Labour costs were calculated using the hourly cost of those involved based on their AfC band and the number of hours they were needed. Other costs included the cost of education delivery, information governance, data ETL, data visualisations and regional and system engagement.

To calculate any potential cost efficiency an appropriate alternative to CLEAR needed to be identified. We used a consultancy alternative with discounted rates exclusive to the public sector through the management consultancy framework. CLEAR roles were aligned with their consultancy roles and their hours needed were converted into days in order to use day rates.

#### Case studies

Case studies of previous CLEAR projects were analysed to estimate the potential long-term return on investment (ROI) of projects.

Each case study looked at the projected benefits of the recommendations from the projects over the next 5 years, using a discount rate of 3.5% per year in line with guidance from the Treasury. Complex change interventions face rates of implementation failure of 30–90%, to account for this a 40% rate of implementation was assumed for the consultancy alternative. 93% of CLEAR associates believe recommendations from CLEAR are more likely to be implemented than those identified by other methods and 86% agreed CLEAR was a more effective way of delivering solutions. We therefore applied an implementation rate of 60% for CLEAR in our base case scenario. As a sensitivity analysis, a range of different implementation probabilities were applied. Savings from solutions implemented were calculated using costs from the Personal Social Services Research Unit (PSSRU) costs of health and social care 2020.

The evaluation was formative as insufficient time had elapsed for all the recommendations to have been implemented. The programme is re-engaging project sites to develop a summative evaluation of the impact of CLEAR.

## Results

### [1] Do CLEAR projects deliver on the value promise and achieve the core aims of the programme?

Interviews highlighted clinicians felt empowered as they believed that CLEAR gave them the opportunity to speak to senior staff in their trusts and be heard. The main findings from the interviews and survey can be found in Table 2.

**Table 2:** Summary of findings from the interviews and surveys

Key points	Description
CLEAR has allowed fellows to gain and practice new skills and knowledge	In particular, the opportunities to develop data skills and speak with managers/directors within the trust was new and empowering. 100% of the survey respondents indicated that, compared to other training on effecting complex change, the CLEAR programme provided a more efficient way of learning and practising skills.
The training CLEAR provides is more relevant to the needs and realities of NHS Trusts	100% of the survey respondents indicated that compared to other training programmes, the CLEAR programme is more relevant to their role and the challenges their team/department face, and 87.5% indicated that, as a consequence, these methodologies are more likely to be adopted. In the interviews, associates indicated that the use of data helped different clinicians within the department build a shared understanding of the problems they faced.
The recommendations generated as a result of the CLEAR programme are more likely to be adopted	87.5% of survey respondents indicated that, compared to other training programmes, the recommendations they developed with clinicians are more likely to be adopted. The interview data showed that, when the fellow was supported well within their department and specialism, and the trust had some existing QI/transformation strategies/ideas, a problem they were keen to tackle, and a reasonable amount of good quality data, the project had a better chance of success.
CLEAR contributes to career progression	62.5% of the survey respondents indicated that the learning and developing activities they completed as part of the CLEAR Programme helped improve their chances of career progression.
CLEAR generates a sense of ‘community’	CLEAR fellows found a sense of community from the face-to-face meetings and enjoyed the opportunity to socialise and talk about their projects informally.
CLEAR fellows report good support from mentors	CLEAR fellows greatly valued the support they received from their mentor. Fellows highlighted the supportive nature of the CLEAR faculty, its willingness to learn and to receive feedback.

In line with the Job Demands-Resources Model (JDRM) [15] CLEAR fed into a greater intention to stay for those working in Trust’s which have taken part in a CLEAR project. The JDRM is a model of workplace health and wellbeing that provides an empirical framework for factors which predict burnout or promote engagement. It explains how the balance between *job demands* (physical, psychological or social aspects of the job which require sustained effort) and *job resources* (physical, social or organisational aspects which function in achieving work goals, reducing job demands or stimulate personal growth) influence retention.

CLEAR feeds directly into the *increase of job resources* by giving NHS staff more autonomy and empowerment in organisational change and transformation. It also provides development opportunities through the training provided. CLEAR also has the potential to significantly *reduce job demands* through more efficient processes which would reduce workload and are more likely to be implemented due to being clinically owned.

### [2] What is the return on investment (RoI) a CLEAR project may bring to a participating NHS organisation and sponsors

The breakdown of labour costs is shown in Table 3, the total cost of each pilot CLEAR project was calculated to be £116,483 (Table 4).

**Table 3:** The labour cost of delivering one CLEAR project

Role	Afc Band	Hours needed	Cost of time needed (£)
CLEAR Associate	7	660	21,594
CLEAR Clinical Sponsor	Consultant	134.4	12,162
Supervisor	8c	247.5	12,726
**Total labour**			**46,483**

**Table 4:** cost of one CLEAR project

	Cost (£)
Education Delivery	12,000
Information governance	8000
Data ETL	20,000
Visualisations	18,000
Regional and System Engagement	12,000
Labour cost	46,482
**Total cost of one project**	**116,483**

There is a cost efficiency of £1.90 for every £1 invested in CLEAR solely from insourcing compared with the consultancy equivalent shown in Table 5. This does not include any other benefits occurring because of CLEAR. It does not account for any solutions that may be implemented because of CLEAR or the improved solutions identified through the use of the data visualisation tool. It also does not include any assumptions around the quality of comparable consultancy solutions or the benefits from the education element of CLEAR which is included in the CLEAR costs.

**Table 5:** Cost of alternative

	SFIA level	Days	Costs^a^	
			Daily	Total
CLEAR associate	3. Apply	82.5	£1100	£108,900
CLEAR supervisor	5. Ensure /advise	31	£1675	£37,800
CLEAR clinical sponsor	6. Initiate /Influence	16.8	£1875	£62,184
Collaboration time	5% of total time	6.5	£12,209	£14,651
**Total Cost external consultancy**			**£223,536**	
**Cost efficiency ratio**			**1.9**	

^a^Daily costs used provided by Economics by Design

### Case studies

#### Case study 1

The CLEAR project led to a recommendation to introduce a frailty unit which is predicted to reduce admissions by 15–25%, avoiding 623–1039 admissions for this department. Assuming a probability of implementation of 60%, this has the potential to lead to a cost-saving of £1.2 m–2.1 m a year. This would result in an ROI of £14.15 for every £1 invested over 5 years compared with the alternative.

#### Case study 2

A recommended change to the Same Day Emergency Care (SDEC) unit could reduce bed days by 889–1502 days, delivering a cost-saving of £452 k per year on average. Assuming a probability of implementation of 60% this would lead to an ROI of £4.54 for every £1 invested over 5 years compared with the alternative.

A subset of the sensitivity analysis around the probability of implementation is presented in Table 6.

**Table 6:** ROI at different implementation probabilities

	Case study 1	Case study 2
ROI when CLEAR 20% more likely to be implemented (base case)	£14.15	£4.54
ROI when CLEAR 5% more likely to be implemented	£4.23	£1.83
ROI when CLEAR 35% more likely to be implemented	£24.07	£7.26

## Discussion

CLEAR is an innovative clinician-led approach to workforce redesign. The key elements of CLEAR that stand it apart from pure process redesign are: (1) the high level of executive and clinical buy in, (2) the legacy of the education that builds intrinsic capacity to continue using the CLEAR approach in practice, (3) and the data driven approach that leads to evidence based recommendations with robust implementation plans and impact metrics increasing. CLEAR has proved so successful that it has been adopted by HEE as a nationally funded programme since 2019 focused on transformation in national and local service priority areas including urgent and emergency care, mental health as well as in the pandemic response. To sustain these transformation initiatives and a national CLEAR faculty is also being established to educate, support and develop a network of clinical leaders.

Increasingly the CLEAR team, working with national and local system partners, is focusing on how the approach can help with a number of key challenges faced by health and social care in the UK as well as globally, over the next decade including;

- Health and well-being of the population – how can local systems improve the health and care outcomes for their populations through more effective prevention and promotion programmes as well as a focus on the wider determinants of health.
- Quality – In addition to looking at the effectiveness of care, improving safety and patient/carer experience are critical. Both are at the forefront of the CLEAR approach through a focus on outcomes with evidence-based improvements.
- Finance – Significant new and additional funding has recently been announced for the NHS and Social Care [16] but many commentators and leaders are concerned that it will be insufficient in the face of demand, backlogs and the demographics of the population [17]. Even the new funding announced brings with it significant productivity and efficiency requirements over the next few years. CLEAR identified over £12.3 million savings in its first seven UEC projects and could help to optimise funding and productivity.
- Workforce – shortages in workforce globally, nationally, and locally are a reality for the foreseeable future and perhaps are the greatest challenge. Workforce redesign and education, including the development of new roles, ensuring professionals time and skills are focused on what they uniquely contribute, are a central part of any solution. Improving the employment experience of future and current employees is also vital. Workforce redesign and improving staff experience are essential elements of the NHS People Plan and CLEAR is seen as one of the solutions.
- Reorganisation of Care –All health care systems in the face of the above key challenges are looking at new models of integrated care, new models of workforce and new models of organising care. How we use best utilise technology and digital innovations to replace, assist and enhance services will be a specific challenge and opportunity. CLEAR is not simply about workforce transformation but transformed models of care.

## Future applications

The next step with CLEAR is the development and embedding of Regional Faculty’s that can deliver CLEAR projects independently. This would build an army of CLEAR competent practitioners who could deliver complex change, eliminating the need for costly externally consultancy. From an educational perspective, the CLEAR team are working with Higher Education Institutions to develop the programme into a formal post graduate qualification e.g. post graduate diploma. The formal recognition of the intensive education and expected outputs from a CLEAR project will support the development of portfolio careers for CLEAR graduates.

To date, the CLEAR approach has only been applied in healthcare. In the future this will be extended and tested in other sectors, such as emergency services and the third sector.

## Conclusions

In summary, CLEAR allows associates to develop valuable new skills in a more productive way - 100% of survey respondents said the CLEAR programme was a more efficient way of learning and practising skills than alternative training. CLEAR provides more cost-effective delivery of complex change programmes than the alternatives – resulting in a cost saving of £1.90 for every £1 spent regardless of implementation success or quality of recommendations. CLEAR recommendations are more likely to be implemented compared to other complex healthcare interventions because of the levels of clinical engagement – and have a potential return on investment of up to £14 over 5 years for every £1 invested.

Finally, CLEAR appears to have a positive impact on staff retention and wellbeing, by giving agency to frontline staff to be involved in the redesign of care– the cost of a CLEAR project is covered if one medical consultant remains in post for a year. By training large groups of individuals in the method we aim to embed the method within the wider healthcare system.

## Supplementary Information

**Additional file 1: Appendix 1.** CLEAR learning objectives. **Appendix 2.** Step by step worked example of the CLEAR methodology. **Appendix 3.** Stages of data processing for a UEC project

## Data Availability

All data generated or analysed during this study are included in this published article.

## Abbreviations

AfC: Agenda for Change
CLEAR: Clinically Led worforcE and Activity Redesign
ECDS: Emergency Care Data Set
EOI: Expression of Interest
ESR: Electronic Staff Record
HEE: Health Education England
KPI: Key Performance Indicators
LSOA: Lower Layer Super Output Codes
MHSDS: Mental Health Services Data Set
NHSEI: National Health Service England and Improvement
NMOC: New Models of Care
PAS: Patient Administration System
ROI: Return on Investment
SEEC: Same Day Emergency Care
UEC: Urgent and Emergency Care
WTE: Whole Time Equivalents
