# Supplementary material for: The clinically led worforcE and activity redesign (CLEAR) programme: a novel data-driven healthcare improvement methodology
Source: BMC Health Serv Res. 2022 Mar 19;22:366. doi: 10.1186/s12913-022-07757-1 (PMC8933657; doi:10.1186/s12913-022-07757-1)
Supplement: Supplementary file 1 — Additional file 1: Appendix 1. CLEAR learning objectives. Appendix 2. Step by step worked example of the CLEAR methodology. Appendix 3. Stages of data processing for a UEC project [file 12913_2022_7757_MOESM1_ESM.docx]

**Supplementary Material**

**Appendix 1: Learning objectives**

This is an example of the Learning Outcomes for the CLEAR programme, showing two learning outcomes from the Clinical Engagement stage for each level of CLEAR practice, from introduction to practitioner. The educational element is the introduction programme, with candidates able to progress up to associate, fellow and practitioner within the CLEAR Faculty.

*Table 1: Learning objectives*

| **Clinical Engagement Stage** | | | |
| --- | --- | --- | --- |
| **Introduction** | **Associate** | **Fellow** | **Practitioner** |
| ***Independently* identify key stakeholders**   - *Contributes to stakeholder list in project plan.* - *Considers the multidisciplinary spectrum of stakeholders in the NHS.* | ***Independently* identify key stakeholders**   - *Contributes to stakeholder list in project plan.* - *Comprehensive mapping of multidisciplinary stakeholders relevant to the specific project.* | ***Independently* identify key stakeholders from all contributing directorates/services or equivalent**   - *Contributes* ***to and confirms*** *an exhaustive stakeholder list for the project.* - ***Filters project specific stakeholder groups*** *into a workable list that is representative of the project.* | ***Independently* identify key stakeholders from all contributing organisations**   - *Contributes to* ***and confirms*** *an exhaustive stakeholder list for the project.* - ***Uses initiative to select influential stakeholders*** *who allow for optimal project impact.* |
| ***Independently* fosters a relationship of open communication and trust with stakeholders and CLEAR faculty.**   - *Completes administrative duties such as dissemination of meeting times, agendas and minutes in a timely fashion* ***with assistance****.* - *Sets meeting agendas* ***with assistance.*** - *Acts as an* ***intermediary for effective communication*** *between stakeholders and the CLEAR faculty.* - *Punctual attendance to engagement activities with stakeholders.* - *Responds positively to stakeholder concern and escalates project issues to appropriate team member.* - *Independently facilitates ongoing dialogue with stakeholders over the course of the project.* | ***Independently* fosters a relationship of open communication and trust with stakeholders and CLEAR faculty.**   - ***Co-ordinates*** *administrative duties such as dissemination of meeting times, agendas, and minutes in a timely fashion.* - *Contributes to meeting agendas.* - ***Initiates communication*** *trails by appropriately identifying when an exchange is needed.* - *Punctual attendance to engagement activities with* ***ability to co-ordinate timekeeping.*** - *Responds positively to stakeholder concern, escalating appropriately and* ***coordinating resolution process****.* - *Independently facilitates ongoing dialogue with stakeholders over the course of the project.* | ***Independently* fosters a relationship of open communication with stakeholders across the organisation.**   - ***Ensures that*** *the administrative duties such as dissemination of meeting times, agendas and minutes* ***are completed by the team*** *in a timely fashion.* - ***Finalises*** *meeting agendas.* - *Initiates communication trails appropriately and flexibly identifies a* ***suitable platform for exchange.*** - *Punctual attendance to engagement activities* ***with a proactive approach to ensure efficient use of time.*** - ***Addresses stakeholder concerns*** *and concerns raised by more junior team members in an appropriate fashion, informing senior CLEAR faculty as appropriate.* ***Deals directly with concerns in their remit, but also recognises limitations and when to escalate.*** - *Independently facilitates ongoing dialogue with* ***senior stakeholders*** *over the course of the project.* - ***Facilitates communication between stakeholders*** *from contributing directorates/services or equivalent.* | ***Independently* foster a relationship of open communication with stakeholders across organisations and between organisations**   - ***Ensures that*** *the administrative duties such as dissemination of meeting times, agendas and minutes* ***are completed by the team*** *in a timely fashion.* - ***Finalises*** *meeting agendas.* - *Initiates appropriate communication on the right platform in a concise manner* ***individualized to the stakeholders need.*** - *Punctual attendance to engagement activities whilst* ***keeping overall oversight to ensure meaningful outcomes relative to time spent.*** - ***Addresses stakeholder concerns a****nd concerns raised by more junior team members in an appropriate fashion, informing senior CLEAR faculty as appropriate.* ***Proactively deals with concerns*** *raised in a systematic manner and competently* ***delegates tasks*** *for swift resolution.* - *Independently facilitates ongoing dialogue with and between* ***senior stakeholders*** *over the course of the project.* |

**Appendix 2: step-by-step worked example of a UEC project**

This worked example is framed around Urgent and Emergency Care. These are not data from a project but represent commonalities that we have observed within our UEC themes.

***Clinical engagement***

The first table shows the outcome of the *clinical engagement* phase, highlighting a key theme with the underlying categories, family codes and supporting quotations. These data are then used to inform fishbone diagrams to determine the root cause of a problem. A section of a fishbone diagram for this theme is shown in the image below.

*Table 2: Key themes, categories and codes, with supporting quotations*

| **Key theme** | **Categories** | **Family codes** | **Example of a supporting** quote |
| --- | --- | --- | --- |
| Frail patients are not having their needs met in the emergency department | 1. Long length of stay in frail patients | - 1. High breach rate of the 4 hour wait target   2. Insufficient estates to review patients in a timely fashion   3. Delayed assessment due to high workload | *“the elderly patients at risk of falls often take ages to get a full assessment- they’re needs are too complex. We really need the frailty team here 24/7” (1.3, 2.2)*  *“it takes ages to get the test results back and then get the medications down from pharmacy. It often means we have to keep people in for longer- especially the frail patients”(2.3, 3.2)* |
|  | 1. Unnecessary admissions of frail cohort | - 1. Under resourced frailty team leading to admissions   2. Lack of social support out of hours   3. Delay in results for diagnostics |  |
|  | 1. Delayed discharges | - 1. referrals miss key information to facilitate discharge   2. Delayed access to medication to safely go home   3. Complex discharges |  |

*Figure 1: Example of a section of a fishbone diagram for the theme above*

Frail patients are not having their needs met in the emergency department

Unnecessary admissions

Under-resourced frailty team

Delay in results for diagnostics

***Data interrogation phase***

Following completion of the clinical engagement phase, associates begin data interrogation by defining they cohort using the filter pages on Tableau^TM^. An example filter page is shown below:

*Figure 2: Filters page*


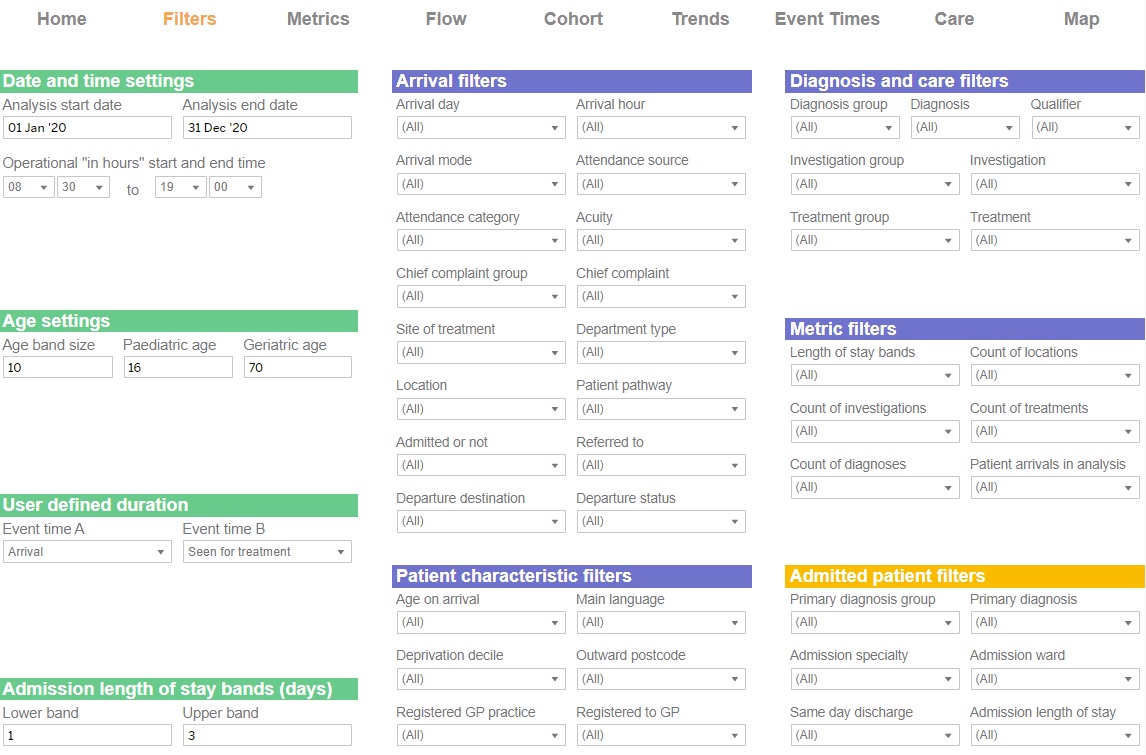


Data can then be visualised in a multitude of ways, to explore, cohorts, trends, flows, diagnostics, investigations, durations, treatments, demand and health inequality. Some examples from the data visualisation tool are show below.

*Figure 3: Sankey diagrams*

Flow through a service can be viewed using a Sankey diagram. The example below shows how all patients flow in the system, highlighting a cohort that attended accident and emergency via ambulance, then were transferred to resus and admitted. This image shows all patients, but we could look at this cohort by cohort.


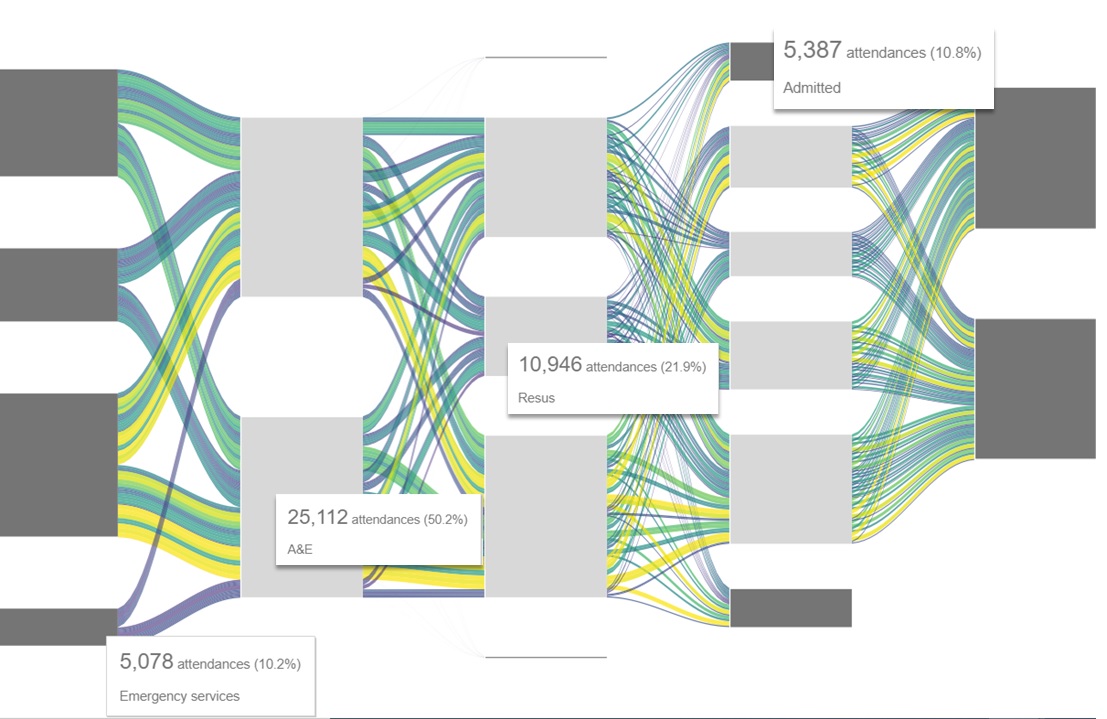


*Figure 4: Heat maps*

Heat maps can be used to identify demand on a service by the time and day, such as time to triage, attendance. This heat map shows us that the busiest time for this department is 8am on a Thursday.


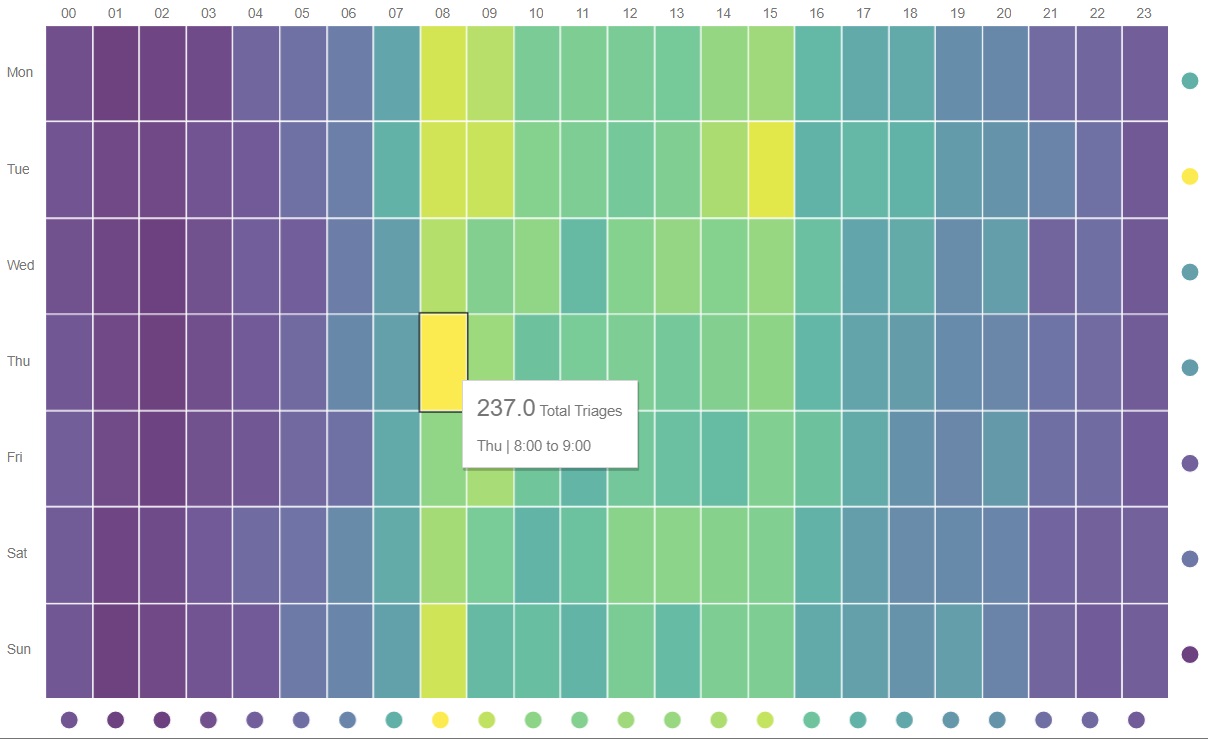


*Figure 5: Care and diagnosis*

Patients can also be viewed by diagnosis, treatment and investigation, for example, it would be possible to view all diagnostics and treatments for patients admitted with chest pain.


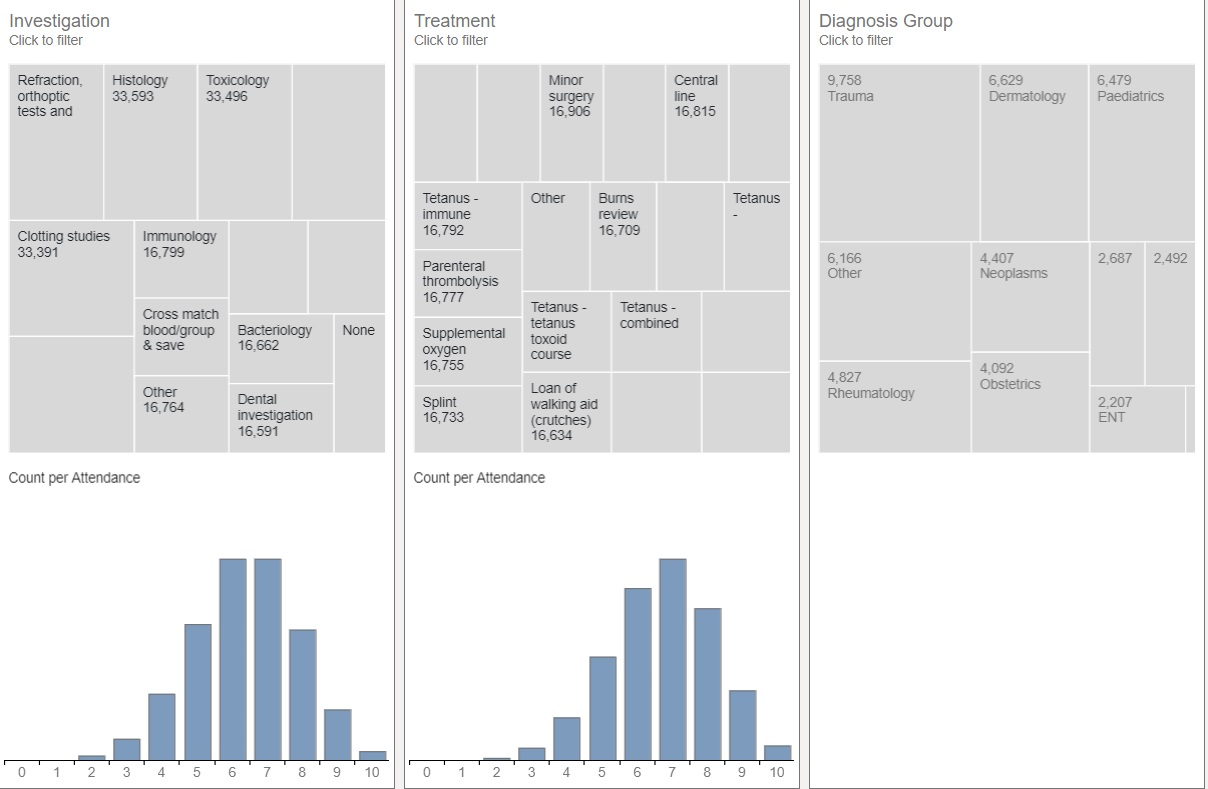


On completion of the data interrogation the qualitative and quantitative data are combined into data triangulation tables linking the key theme with the theoretical framework and supporting data.

*Table 3: Triangulation table*

| **Key theme** | **Theoretical framework/**  **hypothesis** | **Qualitative data** | **Quantitative data** |
| --- | --- | --- | --- |
| Frail patients are not having their needs met in the emergency department | The frailty service design and workforce is insufficient to cope with the number of complex frail patients coming through the service. | *“the elderly patients at risk of falls often take ages to get a full assessment- they’re needs are too complex. We really need the frailty team here 24/7”*  *“I really think if the frailty team had their own space to review patients and were open longer then we could get these patients home sooner and avoid admission”* | - ED sees around 127,000 patients per annum. - 5,715 (4.5%) of those fit the criteria for frailty in 2021. This is around 16 patients/day. - The current frailty team has one consultant (0.3 WTE), one CNS (0.8 WTE) and one therapist (0.5 WTE) and can see around 6-8 patients/ day. - The frailty team works from 8am-5pm. - 40% of admissions for frail patients are between 5pm and 9pm. |

***Innovation stage***

Team used Fresh Eyes approach for idea generation, followed by SWOT analysis to assess each idea. The final ideas to take forward were charted on a desirability versus ease of implementation matrix. The example below shows three ideas generated for this theme. Most CLEAR projects will have multiple recommendations, but one is shown here as an example.

*Figure 6: Desirability versus ease of implementation*


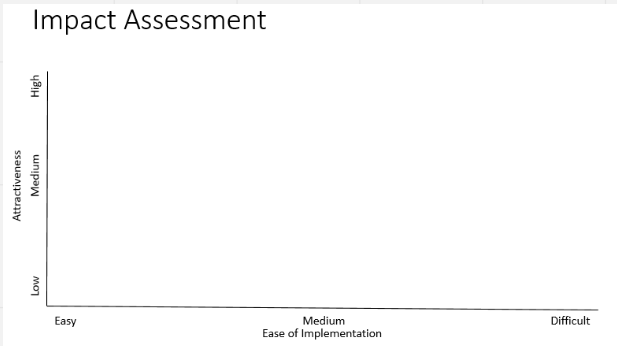


More Frailty Nurses

Extended Frailty MDT with allocated cubicles

Separate Frailty Unit

The solution of extended frailty services with allocated cubicles was selected to take forward and a workforce planned around this. Workforce modelling used data on activity to model the required supply of staff for the extended Frailty team.

*Figure 7: Workforce modelling*

| **FINAL WORKFORCE RECOMMENDATION** |  | |  |  |  |  |
| --- | --- | --- | --- | --- | --- | --- |
|  |  | |  |  |  |  |
| **Staff role** |  | **ACP** | **Band 5 Nurse** | **Physio/OT** | **Specialist Nurse** | **Consultant** |
| **Final WTE** |  | 6.5 | 0.0 | 2.8 | 7.5 | 1.8 |
| **Cost** |  | £163,012 | £0 | £125,752 | £447,119 | £80,024 |
| **Total Cost** |  | £815,907 |  |  |  |  |
|  |  | |  |  |  |  |
|  |  | |  |  |  |  |

***Recommendation stage***

The results and recommendations are reported back to the executive board. This is complete with an implementation plan (Table 4) and a list of recommended performance metrics to evaluate the impact of the NMOC (Table 5).

*Table 4: Example implementation plan*

| Recommendation 1: **Extended frailty service with allocated cubicles**  **Benefits:** Ensure that frail patients are not having their needs following attendance to ED   - - Better provision of multidisciplinary input to improve patient care and ensure that all needs are met   - Improve patient flow through the system and patient experience   - Reduced unnecessary admissions to hospital   - Facilitate early supported discharge   - Minimise 4 hour breach target | | | | | | |
| --- | --- | --- | --- | --- | --- | --- |
| **Sub-components of the recommendation** | **Area** | **Key activities and stakeholders to be engaged / consulted** | **Indicative time scales**. | | | |
|  |  |  | Dec ‘21 | March ‘22 | June ‘22 | Sept ‘22 |
| **1A. Devise criteria to identify appropriate patients**  Consult with the multidisciplinary teams involved in patient care to develop appropriate screening/triage questions | Estate | - Identify allocated cubicles within ED for the frailty service | * |  |  |  |
|  | Workforce | - Support from current frailty team to develop the criteria - Workshop with teams involved in the patient journey to determine implications from the new criteria on patient flow - Implement staff training on new criteria/pathway | * |  |  |  |
|  | Processes & data | - Develop relevant Standard Operating Procedures - Develop and embed referral documentation in IT systems | * |  |  |  |
| **1B. Establish steering group**  The steering group will be comprised of clinicians from across the patient pathway, to review referral criteria and pathway and agree the final workforce for the NMOC | Estates | - Identify necessary equipment required. | * |  |  |  |
|  | Workforce | Steering group should include   - Frailty Consultant - Frailty ACP - Senior ED nurse - Occupational therapist - Pharmacist - Physiotherapist |  | * |  |  |
|  | Processes & data | - Develop and confirm the business case to secure necessary cubicles |  | * |  |  |
|  | General | - Key stakeholder meetings to support the development of the NMOC | * |  |  |  |
| **1C. Secure estates and workforce** | Estate | - Secure location of allocated cubicles - Secure equipment |  | * |  |  |
|  | Workforce | - Confirmation of the workforce requirements for the NMOC - Identify clinical lead |  | * |  |  |
|  | Processes & Data | - Risk assessment - Standard Operating Procedure completed - Confirm launch date for NMOC |  | * |  |  |
|  | General | - Plan launch date, including internal communications to trust staff. |  | * |  |  |

*Table 5: Example of performance metrics.*

| **Recommendation** | **Metric** | **Rationale** | **Review timescale** |
| --- | --- | --- | --- |
| Extended Frailty Service | Number of patients referred to the extended frailty service | Demonstrates clinical need and utility of service, as well as a surrogate measure of the impact on ED | 6 months |
|  | Number patients breaching the 4-hour ED wait target | National KPI and demonstrates the impact of the NMOC on the ED department | 6 months |
|  | Number of admissions for frail patients with a short length of stay i.e. 0-48 hours. | Demonstrate the impact on admissions | 6 months |
|  | Patient experience survey | Receive patient feedback on service | 6 months |

**Appendix 3: Data processing and visualisation worked example for UEC**

1. *Step 1. Data extraction*

Most data extracted for UEC is in Emergency Care Data Set (ECDS) format, this helps to ensure quality and consistency of the data between UEC sites and projects. Further data that is not captured in the ECDS can also be linked to this to augment the analysis.

1. *Step 2: Check for identifiable data*

Data is first checked to ensure that there has not been accidental identifiable data provided in any of the tables.

1. *Step 3: Quality check*

Data tables are then reviewed to ensure that they are complete and usable. Based off the data table schematics, the data is checked to ensure that the Trust have uploaded the correct tables with the correct fields in each table, each field is then checked for:

- Field completion i.e., how many records have been filled in. If data fields are not well populated, then the associates are informed to enable modification of the data analysis strategy or, if the field is vital and the data can be found elsewhere in the hospital system, a new request for updated data can be made
- Data types and format to ensure that it is interpreted correctly in the analysis
- Range of values per field to identify any poorly captured data
- Primary and foreign keys- to spot duplicate records and test table compatibility.

1. *Step 4: Data cleansing*

Often the data provided has fixable small issues such as duplicate records, incorrectly named or partially complete fields, and split tables that need joining together. This is addressed in the data cleansing stage.

1. *Step 5: Data mapping and grouping*

Fields from the raw data tables are mapped to 33n data schemas. This ensures standardisation of field names and data types. During this phase further preparation on the data, ready for analysis is completed, including:

- Replacing national codes with readable definitions
- Replacing long descriptions with clinically relevant short descriptions. This also includes replacing local codes or names for meaningful names e.g., “HPRAU01a” would be replaced by “Rapid Assessment Unit”.
- Grouping values for improved analysis. This can fulfil one of two purposes:
  - Adding grouped values as new additional fields to appear in the analysis. For example, “Presenting Complaint” is grouped to “Presenting Complaint Group”. This allows the clinician to see the high-level types of presenting complaints at a glance, in addition to the more granular data.
  - Grouping many values into more clinically relevant groups to improve the analysis, for example, if there are 10 possible values for “Arrival Method” they can be grouped into “Walk In” and “Ambulance” if the specific transport mode is not required. In this instance, rather than adding a new grouped field, we replace the original 10 values with the 2 grouped values

1. *Step 6. Data transformation*

At this stage the data is reshaped data for visualisation and analysis. This includes:

- Aggregating data to relevant levels of detail
- Calculating dimensions and metrics to show information at the relevant level of detail
- Creating new data tables that contain data for specific visualisations

The output of this stage is a set of final transformed tables in a Structured Query Language (SQL) database, ready for visualisation.

1. *Step 7: Data visualisation*

Using data visualisation software (Tableau^TM^), a workbook is created that allows a user to interrogate the data stored in the transformed data tables. The process for this involves:

- Connecting to the SQL database and building a final data model by linking all the relevant tables using the relevant primary and functional keys to create a relational database model that can interrogated in the final workbook
- Build a suite of worksheets to visualise the data in various ways
- Arrange the relevant worksheets into dashboards to tell the story of the data

The different dashboards in a workbook show information for the same cohort through a different lens. For example, with the filters set to show ambulance arrivals ages over 65, all dashboards in the workbook will show information for this group of patients. One dashboard shows the cohort flow through the emergency department, another shows the trends over time of arrivals and associated performance and care metrics, another contains all the care received during the cohort’s time in the department. Navigating through the various dashboards enables the user to build up a complete picture of the cohort, empowering them with the tools and information to make informed decisions about workforce remodelling and activity redesign.
